# Validity of the portuguese version of the mini nutritional assessment in brazilian elderly

**DOI:** 10.1186/s12877-015-0129-6

**Published:** 2015-10-22

**Authors:** Renata Santos Pereira Machado, Maria Auxiliadora Santa Cruz Coelho, Renato Peixoto Veras

**Affiliations:** Instituto de Nutrição Josué de Castro – INJC, Universidade Federal do Rio de Janeiro – UFRJ, Av. Carlos Chagas Filho, 373 - Ed. do Centro de Ciências da Saúde, Bloco J / 2° andar. Cidade Universitária, Ilha do Fundão, Rio de Janeiro, RJ 21941-902 Brasil; Universidade Aberta da Terceira Idade – UnATI, Universidade Estadual do Rio de Janeiro – UERJ, Rio de Janeiro, Brasil

**Keywords:** Elderly, Malnutrition, Mini nutritional assessment, Validation, Accuracy

## Abstract

**Background:**

Malnutrition is common and affects negatively the health of the older adult. The Mini Nutritional Assessment (MNA), a nutritional assessment tool allows to identify elders malnourished and at risk of malnutrition. The aim of this study is to validate the Portuguese version of the MNA.

**Methods:**

Cross-sectional study with 344 Brazilian elderly. The full version of the MNA was performed, also calf circumference (CC), mid arm circumference (MAC) and body fat (BF). Psychometric evaluation was carried out and correlation, diagnostic accuracy and ROC curves were generated.

**Results:**

Construct validity was supported, all four questionnaire dimensions were evidenced in the Principal Component Analysis and also significant Spearman correlation (*P* < 0.001) were demonstrated. Criterion validity was also evidenced with relevant sensitivity (MAC = 82.8; CI95% = 64.2-94.2) and specificity (CC = 80.0; CI95% = 74.0-85.1). In the ROC curve AUC was excellent (MAC = 0.832; CI95% =0.785-0.873).

**Conclusions:**

The full MNA demonstrated significant results and sufficient exploratory psychometric properties that supported its validity. It seems to be valid tool to access nutritional status of Brazilian elderly.

## Background

Malnutrition is common and affects negatively the health of the older adult. It can lead to various health concerns, including a weak immune system, poor wound healing, muscle weakness and also disinterest in eating or lack of appetite. Malnutrition is often caused by a combination of physical, social and psychological issues. It is more common and increasing in the older population; currently 16 % of those >65 years and 2 % of those >85 years are classed as malnourished. Almost two-thirds of general and acute hospital beds are used by people aged >65 years [1–3]. As the research statistics indicate, not only is malnutrition prevalent in the elderly, it is also frequently misdiagnosed or unrecognized. Many health care professionals are not properly screening or assessing malnutrition in the elderly [2, 4].

The Mini Nutritional Assessment (MNA), a nutritional assessment tool widely used around the world, allows to identify elders malnourished and at risk of malnutrition. It has been translated in over 20 languages with more than 600 PUBMED references [5, 6]. The MNA consists of 18 items including anthropometric, global, dietetic and subjective assessment dimensions. Currently the MNA is used in clinical practice and clinical research [7–10] to assess community-dwelling older adults [11, 12], hospitalized patients [13] or nursing home residents [8, 14, 15].

Studies about malnutrition in the elderly using the MNA in Brazil are insufficient and no validation study has been developed there yet. It very is important to do nutritional assessment in the elderly, making use of valid tools.

The purpose of this article is to validate the Portuguese version of the Mini Nutritional Assessment in Brazilian elderly.

## Method

### Participants

This was a cross-sectional study, conducted with institutionalized elderly residents in public long term geriatric units in Rio de Janeiro, Brazil as part of a larger observational study of nutritional assessment.

Elderly aged 60 year or older were eligible, as recommended by the World Health Organization (WHO) for developing countries such as Brazil [16]. It was also an inclusion criteria have being able to communicate and the strength to carry out an interview and give written informed consent. The exclusion criteria were to suffer from cognitive impairment and not to accept to take part in the survey. The survey consisted of 344 elderly that were residents in one of the 12 municipal shelters in Rio de Janeiro, aged 60–117 years old, 41 % of men and 59 % women and the data were collected in 2001. All included participants provided informed consent.

### Nutritional assessment

The full-form MNA was administered by trained nutritionists, despite the score in the first part of the test. The score range from 0 to 30, and it was calculated as the sum of the values from the 18 items. An MNA score of 24 or higher identifies the patient with a good nutritional status, scores between 17 and 23.5 indicates patients at risk for malnutrition and score less than 17 identifies patients with protein-caloric malnutrition [17].

The anthropometric assessment that were carried out included body weight and height [18], arm span [19], calf circumference (CC) [20], mid arm circumference (MAC) [21] and bioimpedance electric (BIO).

Weight was measured to the nearest 0.1 kg, with the subject in light clothes and no shoes, using a digital scale Kratos with a maximum capacity of 150 kg. Height was measured to the nearest 0.1 cm using a vertical stadiometer Leicester, with the subject’s bare feet close together, back and heels against the wall, standing erect and looking straight ahead. To measure MAC the mid-point between the tip of the acromion and the olecranon process was marked while the subject held the forearm in horizontal position. The measurement was performed on the subject’s arm hanging freely along the trunk with a flexible inextensible tape. CC was measured at the maximal circumference between the ankle and the knee with a flexible tape measure, manipulated to maintain close contact with the skin without compression of underlying tissues. These measures were performed on the non-dominant arm and leg.

In order to classify under nutrition, to BMI it was used the cut-off proposed by the World Health Organization for the elderly [16]. The BMI [weight (kg)/height (m2)] was classified by using the WHO cut-off points, considering women and <23 cm for men, were used to predict under-nutrition [22] and to CC < 31 [16]. To percentage of body fat values the cut-off points were < 24 % for women and < 13 % for man [23].

MAC and CC are parameters used for measurement of muscle mass and subcutaneous adipose tissue [24] and a low MAC among the elderly has been shown to increase risk of mortality and indicates loss of peripheral muscle mass [25, 26]. As for CC, a value of less than 31 cm will indicate muscle loss especially in the lower limb [16]. Body composition was assessed by bioelectric bioimpedance. Fat-free mass, total body fat and per cent body fat were determined.

### Statistical analyses

Descriptive results are presented as means and standard deviations, frequencies and 95 % confidence intervals (CI 95 %). The analysis of data involved descriptive statistics such as mean, standard deviation (SD) and simple frequency. It was used analysis of variance (ANOVA) to compare means between the continuous variables.

To validity it was assessed construct validity and criterion validity, according to Streiner & Norman (2008) [27]. Spearman’s rank correlation coefficients between total MNA score obtained and the criteria of BMI, MAC, CC and BF were calculated. Also measures of accuracy of the tests, sensitivity, specificity, and areas under ROC curves (AUC) were calculated (CI95%). Classification of AUC (range 0–1): acceptable 0.70-0.80, excellent 0.80-0.90, outstanding >0.90 [28].

Exploratory factor analysis with principal components extraction was performed, using PROMAX Rotation with Kaiser Normalization applied to the component matrix.

Significance statistics was considered with p < 0.05. Statistical analyses were performed with IBM SPSS Statistics 19 (SPSS Inc. Chicago IL, USA). Graphics for ROC analyses were created with MedCalc version 12.7.

### Ethics

The local ethics committee of the Federal University of Rio de Janeiro – UFRJ, approved the study protocol. All participants gave written informed consent.

## Results

A total of 344 subjects were evaluated. The full MNA classified 36.1 % of participants in the total data set well nourished, 55.6 % as at risk, and 8.3 % as malnourished. Total MNA scores averaged 22.3 (SD 3.6) and ranged from a minimum of 10.0 to a maximum of 29.0. The age range of the subjects was between 60 and 117 years old with a mean age of 75.4 (SD 9.4) years old.

The socio-demographic profile indicated similarity in the marital status and income of men and women. In relation to age, women have higher prevalence in the older age group and also higher prevalence in the range of education with fewer years of study (Table 1). Nutritional assessment according to MNA is shown in Table 2, with statistical significance for weight, BF, MAC, CC and BMI.

**Table 1:** Socio-demographic and anthropometrics characteristics of subjects according to sex

		Men	Women	Total	*p*-value
		N (%)	N (%)	N (%)	
Age					
	<70	53 (37.9)	53 (26.0)	106 (30.8)	0.013*
	> = 70	87 (62.1)	151 (79.0)	238 (69.2)	
Marital status					
	Married	14 (10.0)	11 (5.4)	25 (7.3)	0.081
	Not married	126 (90.0)	193 (94.6)	319 (92.7)	
Years of Education					
	<=4	70 (50.0)	138 (67.6)	208 (60.5)	0.001*
	>4	70 (50.0)	66 (32.4)	136 (39.5)	
Income					
	<2 minimum wage	98 (90.7)	136 (88.9)	234 (89.7)	0.384
	2+ minimum wage	10 (9.3)	17 (11.1)	27 (10.3)	
MNA					
	Malnutrition	8 (6.6)	17 (9.4)	25 (8.3)	0.242
	At risk of malnutrition	72 (59.0)	96 (53.3)	168 (55.6)	0.246
	Well nourished	42 (34.4)	67 (37.2)	109 (36.1)	
BMI					
	Underweigth	54 (38.6)	54 (26.5)	108 (31.4)	0.012*
	Normal	86 (61.4)	150 (73.5)	236 (98.6)	
MAC					
	Underweigth	8 (5.7)	24 (11.8)	32 (9.3)	0.041*
	Normal	132 (94.3)	180 (88.2)	312 (90.7)	
CC					
	Underweigth	22 (17.9)	56 (29.8)	78 (25.1)	0.012*
	Normal	101 (82.1)	132 (70.2)	233 (74.9)	
PBF					
	Underweigth	29 (24.4)	50 (27.3)	79 (26.2)	0.333
	Normal	90 (75.6)	133 (72.7)	233 (73.8)	

*MNA* mini nutritional assessment, *BMI* body mass index, *MAC* mid-arm circumference, *CC* calf circumference, *PBF* percentage of body fat

* *p* < 0.05, significance level difference between sex (ANOVA)

**Table 2:** Characteristics of nutritional assessment according to the Portuguese version of the MNA

	MNA						
	Malnutrition		At risk of malnutrition		Well nourished		*p*-value
	N	Mean (DP)	N	Mean (DP)	N	Mean (DP)	
Age (y)	25	76,68 (10,49)	168	76,1 (9,15)	109	73,61 (9,52)	0,072
Height (cm)	24	151,70 (8,77)	157	155,40 (10,71)	108	155,62 (10,36)	0,233
Weight (Kg)	24	50,70 (12,37)	157	55,82 (11,80)	108	65,99 (14,79)	0,000*
Body fat (Kg)	22	22,27 (10,96)	149	22,53 (10,17)	104	28,24 (10,12)	0,000*
MAC (cm)	23	25,05 (4,26)	157	26,94 (4,27)	105	29,65 (4,93)	0,000*
CC (cm)	24	30,46 (3,23)	156	32,80 (4,26)	104	35,52 (4,93)	0,000*
BMI (m/Kg2)	24	22,01 (4,89)	157	23,17 (4,79)	108	27,29 (5,75)	0,000*

*MNA* mini nutritional assessment, *BMI* body mass index, *MAC* mid-arm circumference, *CC* calf circumference,

* *p* < 0.05, significance level difference between MNA (ANOVA)

The Kayser-Meyer-Olkin (KMO) measure was 0.64. When above 0.5 it shows an adequation of the method. The Bartletts Test of Sphericity was 623.706, df = 153 and *p* = 0.000, indicating that the sample was adequate for conducting Factor Analysis.

In the Principal Component Analysis of the MNA, the results show a dispersion of the items for 6 components. It explains 52.6 % of the total variance in the explanatory psychometric evaluation. All four dimensions of the MNA are evidenced in the component analysis. The items are arranged according to the dimensions proposed in the original questionnaire, defining the constructs. The anthropometric assessment dimension corresponds to component 1; the global assessment dimension to component 4; the dietetic dimension to component 5; and the subjective dimension to component 2 (Table 3).

**Table 3:** Structure matrix of principal component analysis of the mini nutritional assessment questionnaire variables

Area	Item content	Component					
		1	2	3	4	5	6
Anthropometric assessment	Body mass index	**0,853**	0,140	0,090	−0,015	−0,006	0,066
	Mid-arm circumference	**0,805**	0,070	0,107	0,023	0,078	0,016
	Calf circumference	**0,775**	0,121	0,189	0,054	0,033	−0,032
	Weight loss	**−0,036**	0,720	−0,045	0,079	0,050	−0,095
Global assessment	Independence at home	0,066	−0,070	0,028	**0,127**	−0,077	0,702
	Number of medication per day	−0,169	−0,044	0,008	**0,575**	−0,268	−0,063
	Psycological stress	0,051	0,203	−0,034	**0,523**	0,181	0,154
	Mobility	0,085	0,032	0,698	**0,132**	−0,055	−0,120
	Neuropsychological problems	−0,075	0,346	0,491	**0,125**	−0,032	−0,458
	Pressure skin ulcer	0,129	0,153	0,025	**0,648**	0,114	−0,059
Dietetic assessment	Number of meals per day	−0,127	0,038	0,071	−0,250	**0,374**	0,357
	Serves of high-protein foods	0,009	0,165	0,076	0,051	**0,735**	−0,111
	Fruit and vegetables intake	0,037	0,087	−0,010	0,069	**0,697**	−0,009
	Fluid intake	0,047	0,067	0,319	0,458	**0,272**	−0,464
	Mode of feeding	0,202	0,013	0,663	−0,200	**0,167**	0,166
	Appetite	0,174	0,711	−0,095	0,240	**0,259**	−0,055
Subjective assessment	Self-rated nutritional status	0,181	**0,697**	0,350	0,163	0,097	−0,093
	Self-rated health	0,146	**0,475**	0,356	−0,083	0,059	−0,360

Rotation Method: Promax with Kaiser Normalization

Table 4 shows significant score correlations of the dimensional items of the MNA questionnaire, except for independence at home and number of meals per day.

**Table 4:** Item-total score correlations (Spearman, r) for the Portuguese version of the Mini Nutritional Assessment

Area	Item content	r	*P* - value
Anthropometric assessment	Body mass index	0,468	0,000
	Mid-arm circumference	0,380	0,000
	Calf circumference	0,430	0,000
	Weight loss	0,512	0,000
Global assessment	Independence at home	−0,190	0,746
	Number of medications per day	0,115	0,046
	Psychological stress	0,339	0,000
	Mobility	0,289	0,000
	Neuropsychological problems	0,316	0,000
	Pressure skin ulcers	0,314	0,000
Dietetic assessment	Number of meals per day	0,033	0,563
	Serves of high-protein foods	0,183	0,001
	Fruit and vegetables intake	0,242	0,000
	Fluid Intake	0,326	0,000
	Mode of feeding	0,218	0,000
	Appetite	0,489	0,000
Subjective assessment	Self-rated nutritional status	0,528	0,000
	Self-rated health	0,416	0,000

**Fig. 1:**
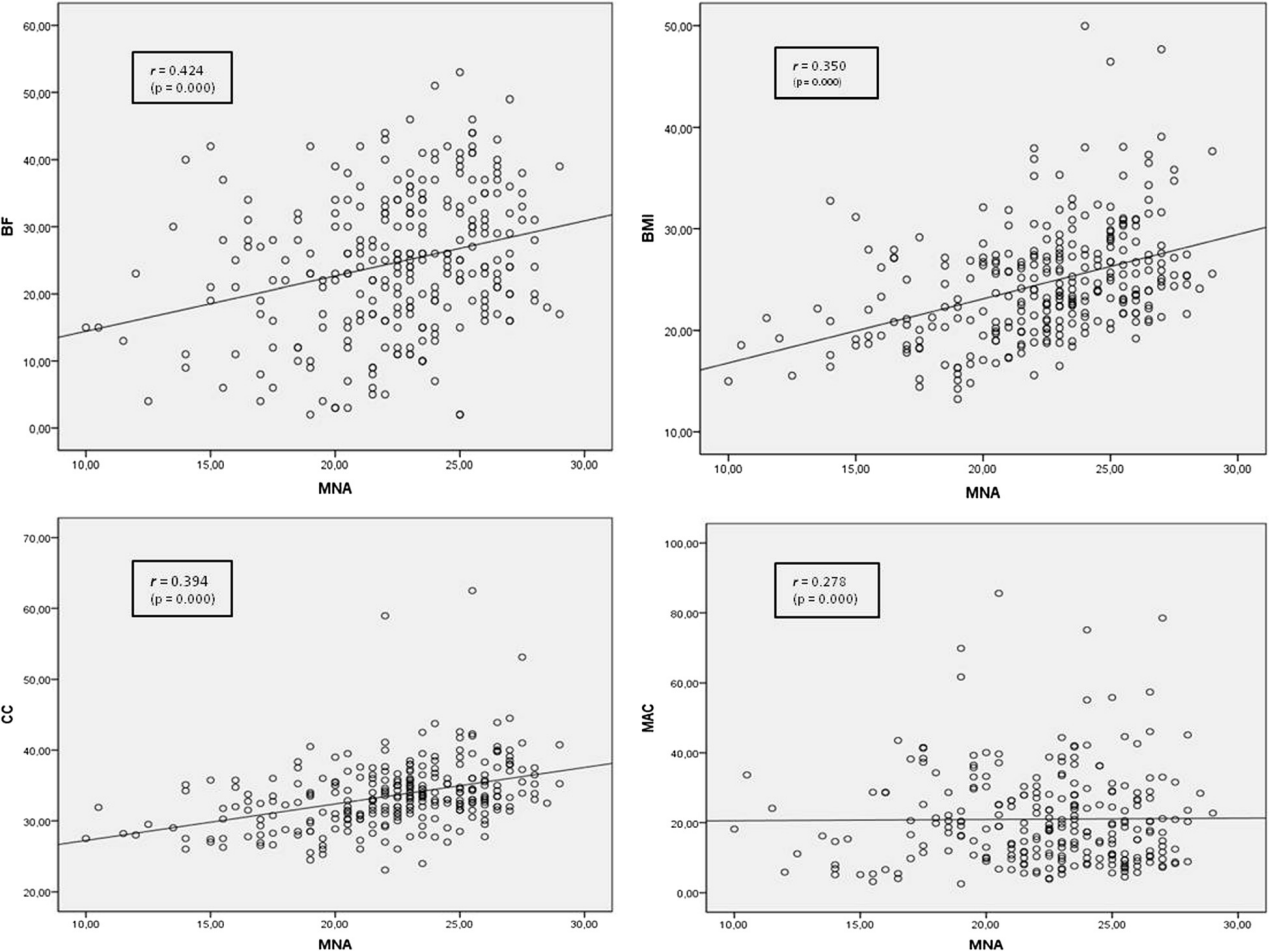
Scatter plot and Spearman Correlation Coefficient of anthropometric measures according to Mini Nutritional Assessment (MNA) score. BMI, body mass index; MAC, mid-arm circumference; CC, calf circumference; BF, body fat; r, Spearman rank correlation coefficients

All nutritional variables had correlation with the full MNA (Fig. 1). There is strong and significant correlation between BF, CC, MAC, BMI and the MNA in this study population.

**Fig. 2:**
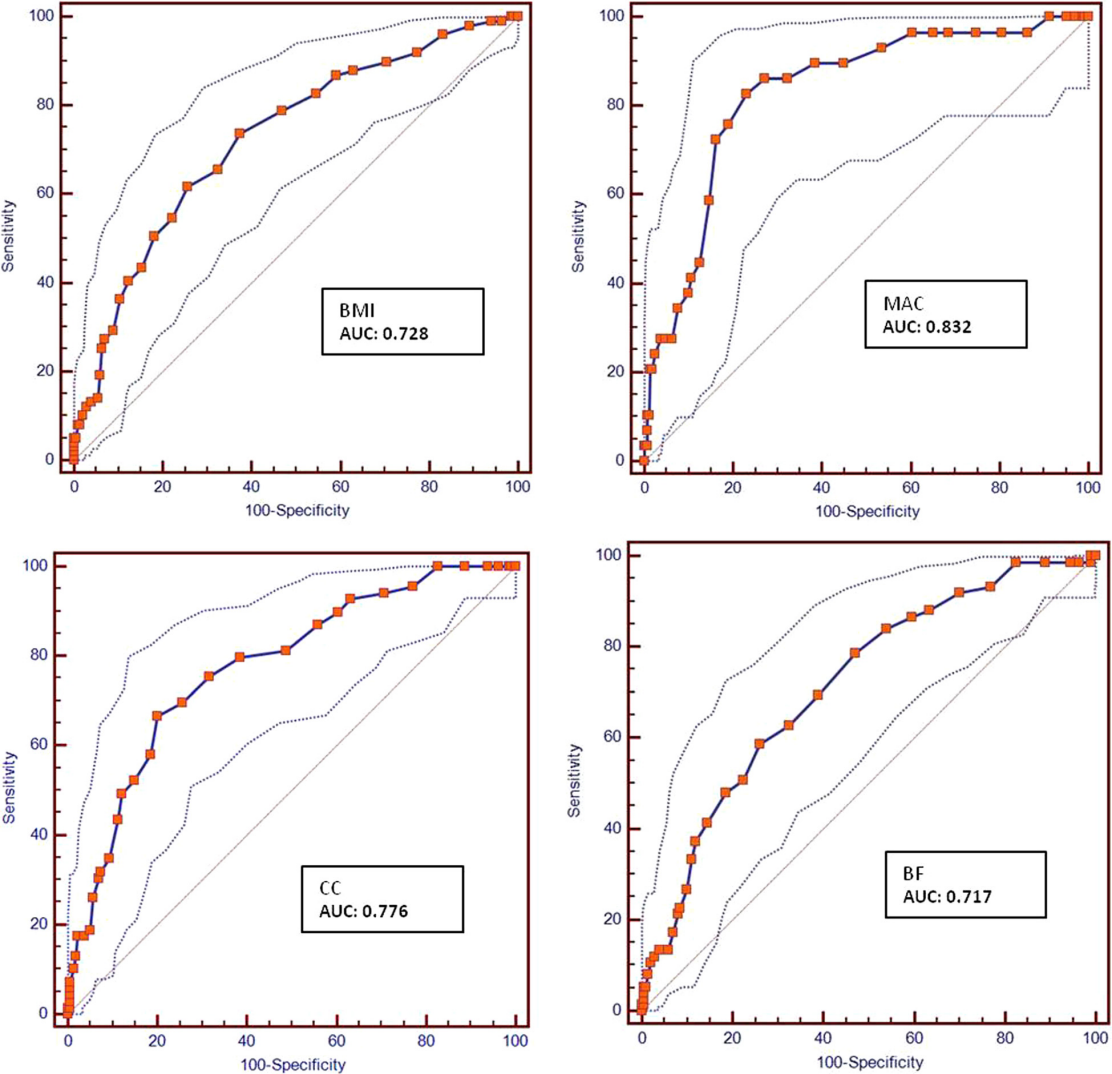
ROC curves of the Portuguese Mini Nutritional Assessment according to anthropometric measures. BMI, body mass index; MAC, mid-arm circumference; CC, calf circumference; BF, body fat; AUC, area under roc curve

The ROC curve is presented in Fig. 2, as well as the corresponding AUC values. In this study, MAC provided excellent discrimination and the other anthropometric measures acceptable discrimination values (Table 5). All indicators showed good sensibility and specificity. MAC was more sensitive (82.8; CI95% 64.2-94.2) and CC more specific (80.0; CI95% 74.0-85.1).

**Table 5:** Accuracy the Portuguese version of the mini nutritional assessment tool according to BMI, MAC, CC and BF

Measure	Sensitivity (95 % CI)	Specificity (95 % CI)	AUC (95 % CI)	AUC discrimination
BMI	73.7 (63.9 – 82.1)	62.6 (55.5 – 69.2)	0.728 (0.674 – 0.777)	acceptable
MAC	82.8 (64.2 – 94.2)	76.9 (71.5 – 81.8)	0.832 (0.785 – 0.873)	excellent
CC	66.7 (54.3 – 77.6)	80.0 (74.0 – 85.1)	0.776 (0.723 – 0.823)	acceptable
BF	58.7 (46.7 – 64.9)	74 (67.3 – 79.9)	0.717 (0.660 – 0.769)	acceptable

*BMI* body mass index, *MAC* mid-arm circumference, *CC* calf circumference, *BF* body fat, *CI* confidence interval, *AUC* area under roc curve, *r* spearman rank correlation coefficients

## Discussion

MNA is used widely around the world to evaluate nutrition status of the elderly. Other studies show that the MNA is an accurate assessment tool for nutritional problems, however it was not validated yet for Brazilian or other Latin American population [17, 29].

In the present study we used anthropometric measures including BMI, MAC, CC and BF. Even though there are not currently, generally accepted criteria for the diagnosis of malnutrition, these parameters have been widely used to evaluate nutritional status [30].

According to these testing results, the MNA full version was shown to have sufficient evidence of validity, including sensitivity and specificity in a sample of older home dwelling people, for identifying elderly hospital at nutritional risk and malnutrition. Anthropometric measures were used as standard to assess concurrent validity and to estimate sensitivity and specificity values.

Validity was supported when testing construct validity, when there is objective criterion that can be used. The Principal Component analysis was robust, with all dimensions represented and with significant correlations. Almost all item-to-total correlations were statically significant. However, not for two of the correlation coefficients: independence at home and number of meals per day. It can be explained by the fact that most of the people in this study gave the same answer, that is, they had the same meals and were not independent at home.

Criterion validity was also supported. It answers the question of how well the scores on a test agree with performance on a task it was meant to predict. The test had significant values of AUC, sensibility and specificity when the criteria BMI, MAC and CC were used. These criteria are common anthropometric measurements and often used in nutritional assessments [31].

According to the American Journal of Nursing, nine studies report sensitivity of the MNA to be 70 % or higher, compared with other nutritional parameters [32–40], similar to this study results. In the original study of MNA as an indicator of protein-calorie under nutrition was found to have a sensitivity of 96 % and specificity of 98 % [17]; however we found lower sensitivity and specificity among Brazilian elderly, but still solid results. Based on the observation of the ROC curve, we also showed that the MNA is accurate.

Some limitations of this study deserve mentioning. First, due to the cross-sectional nature of this study we were unable to estimate a relative risk. Second, in order to increase the possibility of generalization, this association should be studied also in a free living sample. Nevertheless, this study is an important step in supporting scientific using of a popular instrument that measures risk and malnutrition in the elderly.

## Conclusion

In conclusion, various studies support the use of MNA through the world among the elderly population. Malnutrition leads to a decline in health and possibly death; it is often unrecognized and under-treated by healthcare professionals. The full Portuguese version of the MNA demonstrated significant results that supported its validity. MNA has also shown robust exploratory psychometric properties for performing a nutritional screening. It seems to be valid tool to access nutritional status of Brazilian elderly.

## Abbreviations

MNA: Mini Nutritional Assessment
CC: Calf circumference
MAC: Mid arm circumference
BF: Body fat
BIO: Bioimpedance electric
BMI: Body mass index
r: Spearman correlation coefficient
ANOVA: Variance analysis
SD: Standard deviation
AUC: Area under ROC curve
